# Supplementary material for: Stearoyl-CoA Desaturase Is Essential for Porcine Adipocyte Differentiation
Source: Int J Mol Sci. 2020 Apr 1;21(7):2446. doi: 10.3390/ijms21072446 (PMC7177282; doi:10.3390/ijms21072446)
Supplement: Supplementary file 1 [file ijms-21-02446-s001.pdf]

Supporting information

Supplemental Figure S1

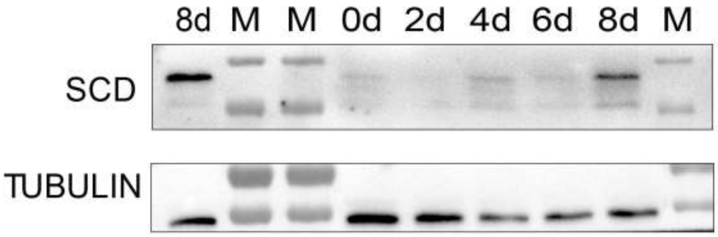

Supplemental Figure S1. The expression of SCD in cells by differentiation day. Western blot analysis results showing that the expression of SCD at differentiation day 8 was higher than it was on differentiation day 0, day 2, day 4 or day 6. The protein marker was named M.

Supplemental Figure S2

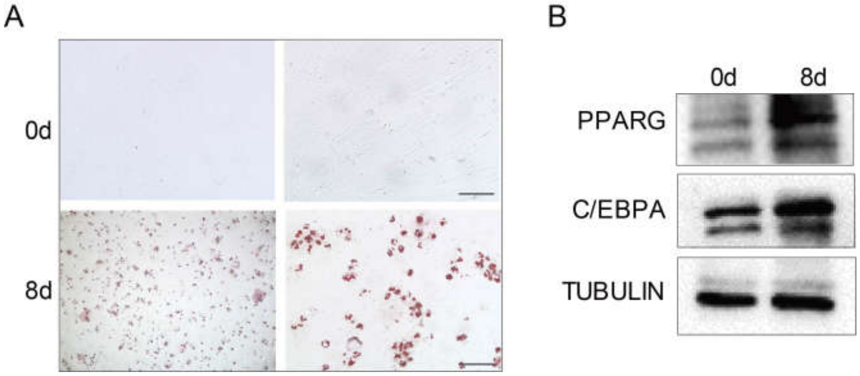

Supplemental Figure S2. Porcine embryonic fibroblasts were differentiated into mature adipocytes. The differentiation method was performed according to a previously described method. (A) Oil red O staining of differentiated day 0 and day 8 porcine embryonic fibroblasts. Red-colored droplets represent mature lipid droplets. Scale bar, 100 µm. (B) The protein levels of the adipogenesis marker genes, PPARG and C/EBPA, were upregulated in differentiated cells.

Supplemental Table S1. Primers used in this study

Gene Name	Forward (5′- 3′)	Reverse (5′- 3′)
ACACA	TCAGAAGGAGGAGGAGGGAA	ATGACGGGACTGTTTGGCTA
C/EBPA	GGCCAGCACACACACATTAGA	CCCCCAAAGAAGAGAACCAAG
CPT1A	ACCAACCCCAACATTCCATA	CCCTTTACCGAAAGTGGTGA
CPT2	ACCTGGACCCTGCATACAAG	CACACACTGGAGAAACTCGC
DGAT1	CCCACCATCCAGAACTCCAT	CGGTCTCCAAACTGCATGAG
DGAT2	CCCTCATAGCTGCCTACTCC	GAGGAAAGACAGGACCCACT
ELVOL6	CCGGAAGTTTGCCATGTTCA	GCAGAAGAGCACAAGGTAGC
FADS2	GGAACCACATCGTCCACAAG	CTTCTTGCCGTACTCAACCG
FABP4	AAGAAGTGGGAGTGGGCTTT	TTCCTGGCCCAATTTGAAGG
FASN	CTGATCAAGGTGCTGCTGTC	CGAAGGAGTTTATGCCCACG

GAPDH	GTCGGAGTGAACGGATTTGG	AGTGGAGGTCAATGAAGGGG
PPARG	CCAGCATTTCCACTCCACACTA	GACACAGGCTCCACTTTGATG
SCD	CTTCCTGATCATTGCCAACA	GCAAACCACCCTTCTCTTTG
SCD #9	AGAAGACATCCGCCCTGAAA	TCTTGCAGGTGGGGATCAAT
SCDKO-GT-large	CACTGCCAGCTCTAGCCTTT	GGCTCCACTATCAGCCCAAG
SREBP1-1C	TTTCTGACCCGCTTCTTCCT	ACGGAACAACTGAGTCACCT
VLCAD	GCCCATCAGAGCATTGGTTT	TAGACAGAACGCAGCCATGA
